# Introductory commentary: a strategic review of options for building on lessons learnt from IMCI and iCCM

**DOI:** 10.1136/bmj.k3013

**Published:** 2018-07-30

**Authors:** Marian Jacobs, Michael Merson

**Affiliations:** 1 University of Cape Town, Faculty of Health Sciences, Cape Town, South Africa; 2 Duke University, Global Health Institute, Durham, North Carolina, USA

## Abstract

Despite advances in child health over the past 20 years, children are still failing to reach their full health and development. **Marian Jacobs** and **Michael Merson** examine what the 2016 strategic review tells about how IMCI and iCCM have contributed to gains in child health, as well as the changes in child health epidemiology, health systems, technology and innovations, and health science

Over the past quarter of a century, child mortality has more than halved.^1^ Yet in 2016, globally, an estimated 5.6 million children died before reaching their 5th birthday, most from conditions that are readily preventable or treatable with proved, cost effective interventions. Millions of others failed to reach their full healthy growth and development.^1^ ^2^ Children need better, more comprehensive care. A third of countries have adopted multisectoral policies to deal with social determinants of child health and promote early childhood development. However, these are rarely institutionalised and fully supported, resulting in fragmented and inadequate services.^3^

Globally and within countries, a number of strategies have been adopted aiming at reducing child mortality and promoting child health. Two decades ago, Integrated Management of Childhood Illnesses (IMCI) was introduced by the World Health Organization and Unicef as a global strategy to “reach all children” with prevention, diagnosis and treatment for common childhood illnesses.^4^ IMCI provides guidance in three key areas: health worker training; health systems’ strengthening; and improving family and community practices.^5^ Since 2010, IMCI has been complemented by Integrated Community Case Management (iCCM), designed to increase access to care for underserved populations, by deploying community health workers to provide diagnosis and management of common illnesses.^6^ IMCI has been adopted in over 100 countries and iCCM has also been widely accepted, mainly in sub-Saharan Africa. In recent years, other global child health strategies have included the Every Newborn Action Plan (ENAP) and Global Action Plan for Pneumonia and Diarrhoea (GAPPD). These work to promote the rights of the child, though few have been as widely adopted and implemented as IMCI and iCCM.

Over the past two decades, the world of children’s health has rapidly changed, necessitating reappraisal of existing child health strategies. The epidemiology of child illness and mortality has shifted, our understanding of aetiologies and determinants has improved and new technologies have emerged. These changes alter the way treatment is delivered. Meanwhile, interest in IMCI has waned at global agencies, and therefore in countries dependent on external funding. Attention has moved to vertical child health areas such as immunisation and/or specific communicable diseases. Thus, IMCI’s uniquely holistic view of child health has arguably been lost. Furthermore, the global child health community now faces a double mandate: the “unfinished agenda” of child survival under the millennium development goals and the broader child wellness agenda advocated by the sustainable development goals and the UN Secretary General’s Global Strategy for Women’s, Children’s and Adolescents’ Health (2016-2030). Beyond ending preventable deaths, the global strategy calls on us to help children “thrive” and to “transform” our social, economic and physical environment to provide conditions enabling each child to reach the highest standard of health.^7^

A fresh look at global approaches to child health is now required.

## Approach of the strategic review

The strategic review of IMCI and iCCM aimed to draw lessons from past measures and current best practices to provide direction on how countries, supported by the global child health community, can deliver the best possible strategies to help each child to survive and thrive. From March to July 2016, the strategic review study team collected and analysed 34 data sources using the following six approaches: literature and desk reviews to combine existing evidence; global key informant interviews to glean insights from experienced professionals; a global survey to provide a detailed overview of IMCI and iCCM application at country level; quantitative analyses of available data to evaluate trends and impact; country case studies to provide a detailed look at implementation; and construction of vignettes of successful child health interventions around the world. The information gleaned from the dataset is both qualitative and quantitative. Collectively, it represents knowledge gained from hundreds of expert opinions and at least 1500 publications related to child health, with contributions from over 90 countries from every continent. Data were analysed iteratively via triangulation between data sources, a structured analysis was undertaken to answer predefined research questions and a 3 day workshop was convened to achieve consensus on key themes. The full methodology of the strategic review is described elsewhere.^8^

This collection of articles (www.bmj.com/child-health ) describes findings from the strategic review. It seeks to provide thoughtful, transparent, evidence based examination of past measures and current best practices, and to consider future needs when rethinking global and national child health strategies. The process of this review, involving iterative analysis of our dataset to extract overarching, high level messages, was designed to aid consideration of some of the most challenging questions in child health. Authors hope to stimulate discussion globally, regionally and nationally, and provide evidence for policy and strategy changes. Continued discussion is necessary to facilitate greater coordination among WHO, Unicef and other global agencies, as well as implementation and funding partners. Such an approach to conception and putting strategies into practice has been lacking recently.^9^

## Findings and insights into improving child health strategies

A descriptive analysis of implementation of the IMCI and iCCM strategies is included to provide context for the following articles, which develop critical themes focused on global child health goals. The concluding commentary explores challenges, opportunities and commitments for improving child health. It is written by WHO and Unicef leaders responsible for developing future global strategies and providing support to national programmes.

Application of IMCI and iCCM has been widespread, but piecemeal within countries.^10^ IMCI has been adopted by over 100 countries and integrated into child health programming in most of these. Countries with the highest mortality rates also have the highest rate of adoption of IMCI. A global survey found that 95% of countries with under-5 mortality rates above 80 per 1000 live births include the strategy in their national child health plans. Implementation has been jointly funded by governments and development partners. The former tend to fund health worker salaries, general programme support, medicines and equipment, and the latter provide the bulk of funding for training and daily subsistence of trainees. Community case management, including iCCM, has also been widely adopted. Over two thirds of countries surveyed reported that community health workers provide care for children under 5. However, for both IMCI and iCCM there are insufficient national and subnational data on the extent or quality of programme adoption and, more importantly, on the effect of the programme.

Moreover, employment of these strategies has not provided a seamless continuum of care between the home, first level facilities and referral facilities, as intended. At country level, implementation of IMCI has focused mainly on the first component (improving health worker skills) to the exclusion of strengthening health systems and community engagement. This, to a large extent, is due to overemphasis on the former by WHO, Unicef and implementing and funding partners.^11^ IMCI was conceived as a “stool with three legs.” However, the failure to provide equal and balanced implementation of all three components has limited its potential effect by undermining built-in synergies (for example, between creation of demand at community level and improved service delivery). Meanwhile, the impact of iCCM has often been constrained by insufficient use of services by target populations. Perhaps this is due to a lack of attention towards activities that would involve and educate communities. Improved integration of these components must be a priority for future child health strategies. This will demand stronger global leadership from WHO and Unicef to make child health a matter of public concern, prioritising those most in need, and putting families, communities and frontline service delivery workers at the centre of activities.

The review identified several obstacles to employing integrated child health strategies–and some potential solutions. District health teams are key to effective operational planning and implementation, particularly as health services are becoming decentralised in many countries. However, district teams must be supported by improved management training, decentralised planning and budgeting, and systems-wide health systems.^12^ Similarly, current global strategies for training and supervision may be limiting, rather than enabling, the action of health workers, owing, in part, to the high cost of training. A meta-analysis of studies of strategies to improve health worker performance is used to pinpoint best practices and outline an initial plan, to be followed by performance monitoring and continual adjustment. Health workers must also be supported by broader health systems improvements, a living wage, and technical and financial assistance from global level.^13^

At the same time, child health practitioners must look beyond the public health facility in child health programming. Although IMCI was designed to focus on improving family and community practices, from breastfeeding to care seeking to sanitation, this component has all too rarely been applied. On the other hand, iCCM focused primarily on case management of the sick child and not holistic care for all children. Increasingly strong evidence (including from randomised controlled trials) is available on the effectiveness of strategies to strengthen the capabilities of individuals, families and communities to improve child health. These include home visits, women’s groups, community discussion and health committees.^14^ However, practice has not matched the increasing evidence, which requires sustained funding and coordination of country-led planning, processes that may be supported by a global re-emphasis on community engagement. Global child health strategies have rarely extended to collaboration with private healthcare providers, even though most children receive private healthcare in many countries. Plans for delivering IMCI and iCCM must establish partnerships and work more effectively with all actors, including private sector healthcare providers, to make sure that every child can access high quality services.^15^

Such improvements in the scope and delivery of interventions will require new ways of thinking, planning, acting and collaborating among all child health stakeholders. Global and country level agencies must work in harmony across programme areas to achieve ambitious child health goals. Global agencies must rationalise and harmonise systems for producing guidelines and include a more diverse set of voices to ensure the plans serve the patient. Future tools to support IMCI, iCCM and other child health initiatives must incorporate promotive, preventive, therapeutic and developmental aspects of child health in a flexible and modular system.^16^ Furthermore, integrated monitoring and evaluation systems may lighten the burden on health workers by optimising existing monitoring strategies and prioritising a small number of indicators, in collaboration with continuing work under the Health Data Collaborative and District Health Information System *2*.^17^ As resources are likely to remain insufficient, global and country level actors must make fairness in child health a primary focus. Thus policies should prioritise intersectoral interventions to deal with the social determinants of health, equity informed planning across countries and measures that reduce the financial burden on poor populations.^18^

## Driving progress in child health

The child health leaders of WHO and Unicef, the two principal United Nations agencies responsible for child health, acknowledge that a lack of unified global leadership has been a major constraint on achieving goals. In the final paper of this collection, they describe the importance of working together, and with all other stakeholders, to provide coordinated support to countries.^19^ They identify key challenges to this commitment: inadequate and uncoordinated funding; insufficient attention to the needs of a particular country; difficulties in integrating new evidence into sound policy and programmes; a multiplicity of fragmented global child health initiatives; and a lack of accountability, internationally and nationally. In response they propose a five point plan: increasing coverage of health interventions through greater multisectoral investment; more versatile implementation tools; enhanced efforts to assess the effect of a programme; more integrated technical and financial support for countries; and greater collaboration on indicators and frameworks to ensure accountability. Moreover, they call for a broader global strategy that covers the sick and well child, from birth to adolescence, that includes promotion, prevention and treatment, and bridges the divide between maternal and child health.

These actions signal a renewed commitment of Unicef and WHO to child health. We urge both agencies to give particular attention to collaborating directly with countries in redesigning, implementing and evaluating the impact of their programmes and in generating the human, financial and infrastructural resources required to ensure that all children survive and thrive. They ask to be held accountable for their actions, and all stakeholders in child health should hold them to this commitment by establishing global targets and monitoring them each year. Today we know more than ever about how to safeguard the health and wellbeing of children. We await strong, intrepid action by global health leaders to translate this knowledge into action.

**Key messages**

- Over the past 25 years child mortality has more than halved, yet an estimated 5.6 million children still die each year, with millions of others failing to reach healthy growth and development
- Child health strategies must be reappraised to account for changing epidemiology, new knowledge about aetiology and effective service delivery, and advances in diagnostic and other technologies
- We introduce a collection of articles describing findings from a strategic review of child health, which aim to provide transparent, evidence based analysis of critical themes focusing on global health goals
- WHO and Unicef express a renewed commitment to child health; both agencies must collaborate directly with countries and ensure sufficient human, financial, and infrastructural resources
- Stakeholders in child health should hold leadership to account to ensure it translates knowledge into action

## Notes

See www.bmj.com/child-health for other articles in the series.

Contributors and sources: This article was written to introduce the findings from a global review of Integrated Management of Childhood Illness (IMCI) and Integrated Community Case Management (iCCM) commissioned by the World health Organization. MJ and MM conceived the outline of this paper, with guidance from *BMJ* editors. Both are responsible for the paper’s intellectual content and wrote and approved the final version for submission. The authors alone are responsible for the views expressed in this article and they do not necessarily represent the views, decisions or policies of the institutions with which they are affiliated.

Provenance and peer review: Commissioned; externally peer reviewed.

This article is part of a series based on findings from a global review of Integrated Management of Childhood Illness (IMCI) funded by the Bill and Melinda Gates Foundation. Open access fees were funded by the Health Systems Research Unit, South African Medical Research Council. *The BMJ* peer reviewed, edited, and made the decision to publish the article with no involvement from the Medical Research Council.
